# Are Deliveries by Inverted T-Incision on the Rise Due to Fibroids?: A Case Report

**DOI:** 10.7759/cureus.24781

**Published:** 2022-05-06

**Authors:** Maryam M Almusalam, Anga Badawi, Nayla Bushaqer

**Affiliations:** 1 Obstetrics and Gynaecology, Royal College of Surgeons in Ireland - Bahrain, Busaiteen, BHR; 2 Obstetrics and Gynaecology, Bahrain Defense Force - Royal Medical Services, Riffa, BHR

**Keywords:** caesarean myomectomies, myomectomy, cesarean section, delivery, pregnancy, fibroid, inverted t-incision

## Abstract

Fibroids are a common finding among women in their reproductive years. In pregnancy, many are incidentally diagnosed and if they are large, they often require careful monitoring concerning their size, number, and location. This case presents a 27-year-old pregnant female with a 15 cm fibroid occupying the lower segment, who had delivered by a cesarean section. This was a complicated delivery conducted at the Bahrain Defence Force Royal Medical Services, requiring a high transverse incision, followed by an extended inverted T-incision. The baby was successfully delivered as breech after prior presenting in a transverse lie. In conclusion, in pregnant cases where large fibroids are obstructing the lower segment, performing a lower segment cesarean section (LSCS) followed by an inverted T-incision is the safest option. Deliveries by inverted T-incisions have increased throughout the years, and this could be explained by the recent favoring of performing cesarean myomectomies, in large obstructing fibroids. Nowadays, with advanced preparations, myomectomies during cesarean section are safely and frequently performed when the benefits outweigh the risks.

## Introduction

Fibroids are benign fibro-muscular growths in/around the uterus, also referred to as myomas/leiomyomas. Their incidence increases with age from 40% at the age of 35 years up to 80% by the age of 50 years, where they can be symptomatic in around one in three females. Symptoms such as heavy painful periods, abdominal pain, dyspareunia, and lower back pain are commonly reported. Fibroid growth is often affected by high levels of estrogen; therefore, they are generally present during reproductive years and tend to shrink at menopause [1].

During pregnancy, they can cause preterm delivery, delivery by cesarean section, and rarely, miscarriages [1]. It is crucial to monitor fibroids, as they may increase in size in up to 70% of the cases during the first and second trimesters, leading to unpredictable outcomes, especially during labor, in terms of mode of delivery and the fetus’s wellbeing [2]. They grow in different sizes, numbers, and locations, leading to different challenges throughout pregnancy. This case is a report of a large lower segment fibroid limiting the possibility of a lower segment cesarean section (LSCS) and eventually allowing the delivery of the baby by inverted T-incision followed by a myomectomy. 

## Case presentation

A 27-year-old female, G3P2, presented to the emergency department (ED) at five weeks gestation with vaginal bleeding associated with mild abdominal pain. The patient had neither associated symptoms such as nausea, vomiting, fever, or chest pain, nor had contact with any COVID-positive patients. There was a negative history of chest pain, dyspnea, hospitalization, and recent travel. She had neither known allergies nor any known chronic medical conditions, except for an interstitial fibroid of around 4.7 cm. The fibroid was incidentally discovered during her second pregnancy, four years ago, in the right lateral wall of the lower uterine segment. Although the fibroid did not cause any complications during that pregnancy, she experienced gestational diabetes mellitus which complicated the pregnancy, furthermore, resulting in a pre-term delivery at 30 weeks gestation and a birth weight of 2.8 kg. Both previous deliveries were spontaneous vaginal delivery (SVD) with the first being full-term. Her menstrual cycle was regular, however always heavy, and prolonged, so she was managed by progesterone pills. All her cervical pap smears were regularly conducted as per schedule, with negative results for malignancy. She had previously used an intra-uterine contraceptive device (IUCD) for both contraception and menorrhagia, with no other forms taken. Upon examination, she was vitally stable, body mass index (BMI) of 25.67 kg/m2, with a normal general and abdominal examination. She was in mild pain, therefore, oral paracetamol tablets were given and the vaginal bleeding was managed by dydrogesterone 10 mg tablets that were taken twice daily until the bleeding stopped.

At seven weeks gestation, the ultrasound scan showed a single fetus in addition to an anterior wall fibroid of 8 cm x 8 cm in size in the lower uterine segment, which had increased in size to 10 cm x 10 cm by the 11th week of gestation. By week 15 of gestation, the placenta was anterior, and away from the fibroid, however, areas of cystic degeneration were apparent within the fibroid. Increased vascularity was seen in the right lateral wall, encroaching on the internal cervical os. Incidentally, a hyperechoic lesion of a suspected hemangioma of 1 cm was noted in the right hepatic lobe.

At 21 weeks gestation, a detailed scan was repeated, which did not show any significant changes to the fibroid, with no abnormalities detected in the fetus. The right pelvicalyceal system was revealed to be mildly dilated by 27 weeks gestation, which could have been worsened by the gravid uterus along with the fibroid. At 32 weeks gestation, the fetus was lying in a breech position. She was then scheduled for LSCS at 36-37 weeks gestation. The patient was referred to the Radiology Department for MRI to localize the borders of the fibroid before surgery. It appeared that it was a 15 cm x 15 cm x 13 cm submucosal fibroid, occupying the entire lower segment, covering the cervix completely, with an extension into the upper segment, which was not completely visible by ultrasound.

**Figure 1:**
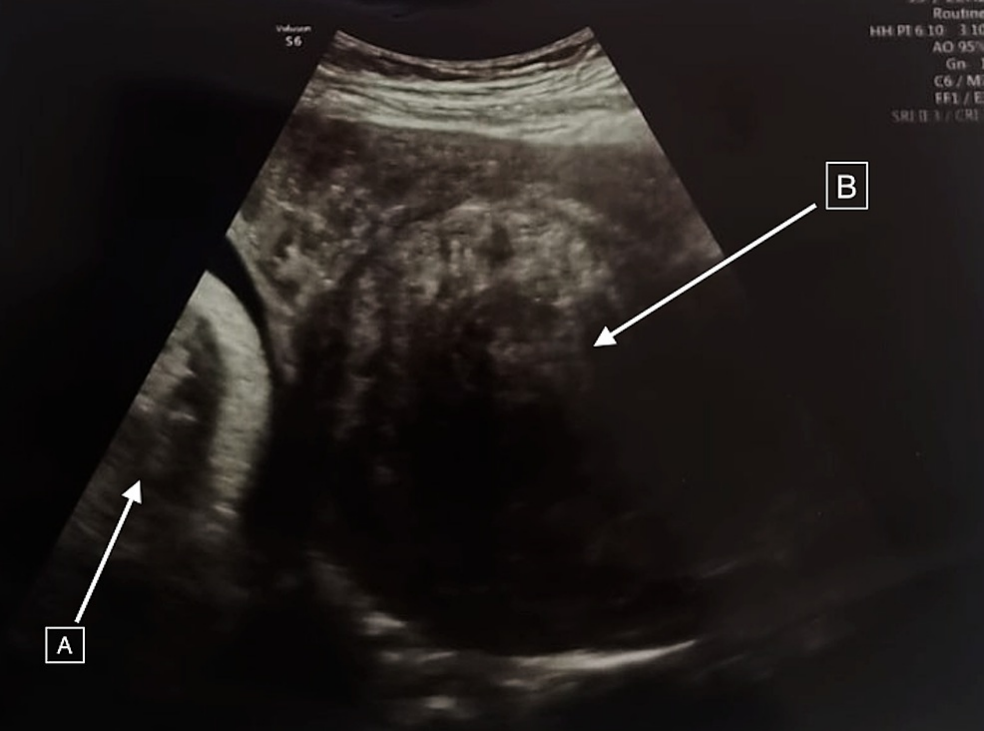
An abdominal ultrasound showing: A. The fetus’s head. B. The obstructing fibroid.

**Figure 2:**
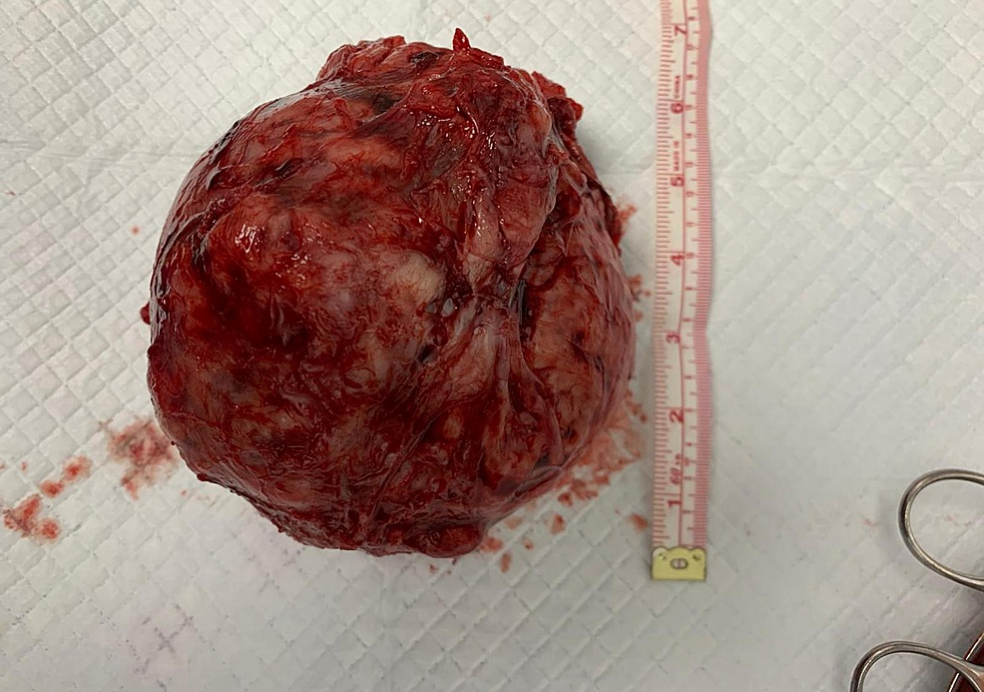
Fibroid post-myomectomy; around 6 in (15 cm) in size.

An early delivery date at 36 weeks gestation was advised, to avoid the possibility of the patient going into labor in the later weeks, along with its complications. The fetus was lying in a transverse position and delivery was performed by a transverse incision in the uterus, just above the fibroid, into the upper segment. This was extended by an inverted T-incision and the baby was delivered breech with a weight of 2.4 kg. Following delivery, a myomectomy was performed; mainly due to the obstructing nature of the fibroid, and the fibroid was successfully removed (Figures 1-2). The histopathology report confirmed the uterus mass as a leiomyoma weighing 871 g, in addition to some ischemic changes in the placenta. Intra-operatively, the patient lost around 1 L of blood during the combined procedure of LSCS, inverted T-incision, and the myomectomy. Some two units of packed red blood cell (RBC) were given.

Nine days post-op, she presented to the ED with low-grade fever and mild pain at the surgical site, which was managed by oral amoxicillin/clavulanate potassium for a one-week duration, with oral paracetamol when needed, and local antiseptic spray. Two days following this, she presented to the ED with mildly unresolved pain. This was managed symptomatically along with wound care only since the lab results showed no signs of infection and the patient was soon discharged. She was advised to have an 18-month inter-pregnancy gap, and for future pregnancies, vaginal birth after cesarean (VBAC) would not be possible due to the inverted T-incision, leaving cesarean section (CS) as the best choice. 

## Discussion

Lower segment fibroids often result in a burdensome delivery, yet many published cases were nevertheless, managed by a lower segment CS [3-4]. The patient’s fibroid was occupying a large portion of the lower segment which extended into the upper segment. It is advisable to avoid the fibroid when performing a CS, due to the high vascularity during pregnancy, and increased risk of hemorrhage. The obstructing nature of the fibroid has led the fetus to remain in a transverse lie, which in turn led to an impossible vaginal delivery. An upper segment transverse incision, in this case, was most suitable to be performed, as it favors the delivery of a transverse lying fetus as well as avoids the highly vascular fibroid from bleeding. Transverse lie occurs in around <0.5% of all pregnancies, and in less than 8% a transverse incision is converted to an inverted T-incision [5], as in our case, due to the insufficiency of reaching the uterine cavity and issues with extraction. Therefore, in concordance with the guidelines, a further extension was performed by an inverted T-incision, and the fetus was rotated vertically and delivered as a breech.

According to the published literature, the above steps of converting from transverse to inverted T-incision were proven to be the best option when facing a difficult delivery, especially when considering blood loss and in preterm deliveries, as in our case, where the myoma was the challenging factor. To prevent injuries of enormous stress to the fetus, breech delivery was most suitable. Studies have also suggested, that in cases where an inverted T-incision is not applicable, a vertical incision or a J- shaped incision are other options to consider [6-7]. Inverted T-incisions incidence has increased from 0.2% to 0.9%, with a remaining high risk to maternal health [8]. There is no clear reason why the use of this incision has increased, however, we believe that this could be explained by mainly the wide availability of blood products for resuscitation and the acceptance of performing myomectomies during CS in selected patients. Another reason could also be due to the risks that follow alternative incisions such as the classical CS, as the closure of such incisions is very difficult and pose a threat to future pregnancies. Uterine rupture occurs up to 9% in those who have undergone the classical CS as opposed to the 1.5% observed in those who had a lower transverse incision [9]. Classical CS is not superior to performing a transverse incision converted to an inverted T, as they carry a high perinatal and maternal morbidity risk, especially in terms of blood loss. The article has further noted that despite the equal amount of blood loss in both groups, higher rates of transfusion and postoperative infection were more significant in classical cesarean rather than in inverted T and transverse incisions separately [8].

After the successful delivery of a healthy baby, a myomectomy was performed. Whether or not performing myomectomies during CS is advisable was debatable in the past. Despite the increased risk of bleeding intra-operatively, we believe that favoring myomectomies, in cases with large, symptomatic fibroids, which interfere in normal term deliveries, in young healthy females may be a reasonable decision, with an added benefit of avoiding a future operation. Studies [10-11] have concluded that with appropriate hemostatic techniques, cesarean myomectomies should be preferred over CS alone, especially with large, multiple fibroids. Goyal et al. [10] have also noted that despite higher rates of blood transfusions in those who have undergone cesarean myomectomies, results show insignificant results regarding longer hospital stay, hemorrhage, and fever among both groups.

Myomectomies during cesarean sections are both safe and sensible as they are far more common procedures nowadays, especially when the benefits outweigh the risks [3-4]. Klatsky et al. studied the patterns of uterine fibroid effects on pregnancy outcomes. They noted that fetal malpresentation was 2.5-fold higher in patients who had fibroids. Preterm deliveries were 16% higher among the affected group with fibroids larger than 5 cm in size, which tend to deliver at around 36 weeks gestation [12], as in our case. Considering the complications of retaining fibroids during pregnancy [1, 12], we believe that fibroids that tend to massively grow during pregnancy need to be considered for removal, especially if a CS has been scheduled. The complications of reserving such fibroids can negatively affect the viability of future pregnancies [13]. Fibroids also tend to cause symptoms such as pelvic pain and menorrhagia, which this patient experienced. It is, therefore, more beneficial to remove them to avoid future myomectomies or hysterectomies in the absence of any oncological reasons [13-14].

## Conclusions

In conclusion, inverted T-incisions are infrequently performed in pregnancies as a method of delivery. However, with an increased preference for performing myomectomies during CS, when a lower segment incision is insufficient, inverted T-incisions are considered the next option. Deliveries involving large obstructing fibroids could explain the rise in the use of inverted T-incisions. It is, therefore, important to highlight that when LSCSs are not possible, it is safer to perform an inverted T-incision over the classical incision. Myomectomies are generally safely performed during CS nowadays, with benefits exceeding the risks, due to the improved preparation pre-/intra-/post-operation which mainly involves the availability of blood transfusions.

## References

[1] (2022). Overview fibroids. https://www.nhs.uk/conditions/fibroids/..

[2] Pergialiotis V, Sinanidis I, Louloudis IE, Vichos T, Perrea DN, Doumouchtsis SK (2017). Perioperative complications of cesarean delivery myomectomy: a meta-analysis. Obstet Gynecol.

[3] Garg P, Bansal R (2021). Cesarean myomectomy: a case report and review of the literature. J Med Case Rep.

[4] Ghaemmaghami F, Karimi-Zarchi M, Gharebaghian M, Kermani T (2017). Successful myomectomy during cesarean section: case report &amp; literature review. Int J Biomed Sci.

[5] Visconti F, Quaresima P, Rania E (2020). Difficult caesarean section: a literature review. Eur J Obstet Gynecol Reprod Biol.

[6] Dalvi SA (2018). Difficult deliveries in cesarean section. J Obstet Gynaecol India.

[7] Takeda J, Ishikawa G, Takeda S (2020). Clinical tips of cesarean section in case of breech, transverse presentation, and incarcerated uterus. Surg J (NY).

[8] Patterson LS, O'Connell CM, Baskett TF (2002). Maternal and perinatal morbidity associated with classic and inverted T cesarean incisions. Obstet Gynecol.

[9] Kan A (2020). Classical cesarean section. Surg J (NY).

[10] Goyal M, Dawood AS, Elbohoty SB, Abbas AM, Singh P, Melana N, Singh S (2021). Cesarean myomectomy in the last ten years; A true shift from contraindication to indication: a systematic review and meta-analysis. Eur J Obstet Gynecol Reprod Biol.

[11] Huang Y, Ming X, Li Z (2020). Feasibility and safety of performing cesarean myomectomy: a systematic review and meta-analysis. J Matern Fetal Neonatal Med.

[12] Klatsky PC, Tran ND, Caughey AB, Fujimoto VY (2008). Fibroids and reproductive outcomes: a systematic literature review from conception to delivery. Am J Obstet Gynecol.

[13] O' Sullivan R, Abder R (2016). Myomectomy at the time of cesarean delivery. Ir J Med Sci.

[14] Di Cello A, Borelli M, Marra ML (2019). A more accurate method to interpret lactate dehydrogenase (LDH) isoenzymes' results in patients with uterine masses. Eur J Obstet Gynecol Reprod Biol.

